# The Contribution of Minor Cereals to Sustainable Diets and Agro-Food Biodiversity

**DOI:** 10.3390/foods12183500

**Published:** 2023-09-20

**Authors:** Laura Gazza, Francesca Nocente

**Affiliations:** CREA–Research Centre for Engineering and Agro-Food Processing, Via Manziana, 30-00189 Rome, Italy; francesca.nocente@crea.gov.it

Since the second half of the 20th century, the intensification of agriculture by increasing external inputs (fertilizers, pesticides), cropland expansion, and the cultivation of only a few selected cereal species or varieties have caused the loss of biodiversity and ecosystem services on farmland. As a result, at present, only three major cereals—wheat, rice, and corn—dominate agriculture and human sources of nutrition on a global scale, whilst many traditional cereal species, varieties, and landraces have gradually disappeared from fields, or their cultivation is limited to a small scale. However, the need for sustainable agriculture in the context of climate change has sparked interest in ‘minor or underutilized’ cereals that can be utilized on a global level. These cereals, some of which still represent the main staple food at the national or regional level, include einkorn, emmer, millet, oats, rye, spelt, sorghum, teff, triticale, and tritordeum. Indeed, despite their low yield, minor cereals have proven to be inherently resilient and rustic and are therefore able to withstand adverse climatic conditions and are well suited to grow under low-input cultivation management on marginal lands; thus, they have less negative impacts on the environment. Due to the increased demand for healthy, nutritious, non-conventional, and sustainably produced food, minor cereals have re-gained the attention of researchers, farmers, producers, and consumers. Indeed, as a source of carbohydrates, proteins, vitamins, minerals, fiber, and antioxidant compounds, their inclusion into daily diets can reduce the risk of chronic diseases such as cancers, type II diabetes, obesity, and cardiovascular diseases. To ensure their sustainable production on a large scale in the future, it is absolutely essential to strengthen research to ascertain the genetic variability of minor cereals and select genotypes with promising traits for yield, disease resistance, climate resilience, and nutritional quality. Research should also be directed toward optimizing agronomic practices and developing technological processes able to produce innovative foods, preserve nutrients and bioactives, and meet consumer expectations. In this Special Issue, twelve papers (including ten original research articles and two reviews) address the topic of ‘Sustainable Diets and Agro-Food Biodiversity’, investigating the possible contribution of minor cereals to tackle the future demand for healthy and sustainable food. Among minor cereals, sorghum was the most investigated, probably due to its ability to grow in semi-arid climates, its nutritional characteristics, as well as its being a gluten-free cereal. Robles-Plata et al. [1] analyzed the biophysical, nutraceutical, and techno-functional properties of pigmented sorghum (red and yellow) and popcorn (blue, purple, red, black, and yellow). Besides differences in biophysical and proximate composition among species and varieties, sorghum exhibited higher total phenolic content, whereas higher total anthocyanin content was found in the purple, blue, and black popcorn, as well as in red sorghum.

In a study by Renzetti et al. [2], the physical and sensory properties and consumers’ perceptions of bread obtained from a flour blend of sorghum, cassava, and cowpea were assessed. The overall results suggest these African resilient crops have potential as an alternative to wheat in bread-type products, especially in Sub-Saharan Africa.

Sorghum was also investigated in a research article by Mawouma et al. [3], which analyzed the nutritional and phytochemical profiles and antioxidant activity of sorghum and pearl millet local varieties that are regularly produced and consumed in Cameroon. Thus, the most promising cultivars of sorghum and pearl millet to tackle nutrient deficiencies and non-communicable diseases were identified.

Banu and Aprodu [4] performed a comparative study of the physicochemical and functional properties of seven gluten-free flours obtained from cereals (sorghum, rice, oat, and foxtail millet) and pseudocereals (amaranth, quinoa, and buckwheat), as well as the thermo-mechanical properties of their dough.

Pontieri et al. [5] compared both the chemical composition and the content of fatty acids and minerals of three sorghum varieties that differed in pericarp color (white, red, or black) grown in the Mediterranean basin. Results indicated that grain pericarp color is associated with unique nutritional profiles and that the sorghum varieties developed for commercial production in the USA were also suitable to be grown in the Mediterranean environment.

Another example of using minor cereals for functional food is reported in a paper by Živkovic et al. [6] that explored the effect of germination on the secondary metabolite composition in spelt grains. According to the results, germinated kernels showed a significant increase in the total phenolic content of several secondary metabolites, as well as antioxidant activity, especially after 96 h of germination, indicating this biotechnology could be used in a strategy to enhance cereal health-promoting effects.

In a study by Gazza et al. [7], the flours of two einkorn varieties, obtained by an ultra-fine milling process, were used to produce wholewheat dry pasta. The technological, nutritional, and sensorial characteristics of einkorn spaghetti were assessed, and results indicated that, despite the very weak gluten network, einkorn proved to be a viable alternative cereal to durum wheat to produce dry pasta.

In a research article by Nocente et al. [8], two ancient Caucasian hulled wheats grown in Italy, Triticum timopheevii and Triticum zhukovskyi, were analyzed for physical, nutritional, and technological characteristics. Both Caucasian species had high protein content and antioxidant activity and good technological and rheological performances, suggesting these wheats can serve as a promising raw material for the formulation of flatbreads, biscuits, and pasta.

The article by Onyango et al. [9] compared the impact of native, steamed, or malted finger millet and amaranth seeds on the rheological properties of dough and the physicochemical quality of composite breads. As per their findings, malting and steaming appear to be promising approaches for amaranth to improve composite bread quality, whereas, with respect to finger millet, the treatments were not suitable to improve its breadmaking potential. 

Spaggiari et al. [10] characterized three common wheat evolutionary populations (EP), a cultivation technique characterized by mixing and sowing many wheat genotypes together to allow the crop to adapt genetically over several years in relation to specific pedoclimatic conditions. The nutritional, chemical, and sensory qualities of three different breads obtained using organic EP flours that were produced following a traditional sourdough process were investigated. Although the technological quality of EP flours seemed unsuitable for bread-making, sourdough baking allowed excellent workability of the EP doughs and good structure of the loaves.

The topic of the role of minor cereals in achieving the goal of food sustainability and diversity was also addressed in a comprehensive review by Majzoobi et al. [11]. In their review, the authors report previous knowledge published on ancient cereals and pseudocereals in terms of physicochemical properties, nutritional profile, and food industry applications and the comparison with modern crop counterparts. The review also discusses the opportunities and challenges of ancient cereals as useful crops to address Goal 2: Zero Hunger—United Nations Sustainable Development Program, as well as the issue of malnutrition globally, providing a guide for decision-makers and policies to face these threats. 

In a more targeted way, the review of Gowda et al. [12] focuses on millet, one of the major underutilized, highly nutritive food crops. The review provides an overview of original research articles and reviews that highlight the nutritional characteristics of Indian millets (foxtail, kodo, proso, little, and pearl millets) and the effects of primary (dehulling, soaking, germination, drying, polishing, and milling) and secondary (fermentation, germination, extrusion, cooking, puffing, popping, malting, baking, flaking, and extrusion) processing techniques on the nutritional features of this cereal. Germination and fermentation improve the overall nutritional characteristics of millets, whereas excessive dehulling, polishing, and milling reduce dietary fiber and micronutrients. This overview on millet can help encourage farmers, the food industry, researchers, and consumers in millet utilization and in selecting suitable processing techniques to optimize nutrient value, increase the bioavailability of nutrients, and help combat food and nutrition security.

In conclusions, this Special Issue provides a fundamental understanding of the current strategies for the revitalization of underutilized cereals, which represent a reservoir of biodiversity that is useful to ensure sustainable production and food security in the context of climate change.
